# A Ni-Doped Carbon Nanotube Sensor for Detecting Oil-Dissolved Gases in Transformers

**DOI:** 10.3390/s150613522

**Published:** 2015-06-09

**Authors:** Jia Lu, Xiaoxing Zhang, Xiaoqing Wu, Ziqiang Dai, Jinbin Zhang

**Affiliations:** 1 State Key Laboratory of Power Transmission Equipment & System Security and New Technology, Chongqing University, Shapingba District, Chongqing 400044, China; E-Mails: jia-lun@163.com (J.L.); xiaoqingwu@cqu.edu.cn (X.W.); 2 State Grid Guizhou Liupanshui Power Supply Company, Liupanshui 553000, China; E-Mail: daizq382812@163.com; 3 Chongqing Power Company, Beibei 400700, China; E-Mail: wufeng.200809@163.com

**Keywords:** oil-dissolved gas, carbon nanotubes, nickel-doped, sensor response, linear relationship

## Abstract

C_2_H_2_, C_2_H_4_, and C_2_H_6_ are important oil-dissolved gases in power transformers. Detection of the composition and content of oil-dissolved gases in transformers is very significant in the diagnosis and assessment of the state of transformer operations. The commonly used oil-gas analysis methods have many disadvantages, so this paper proposes a Ni-doped carbon nanotube (Ni-CNT) gas sensor to effectively detect oil-dissolved gases in a transformer. The gas-sensing properties of the sensor to C_2_H_2_, C_2_H_4_, and C_2_H_6_ were studied using the test device. Based on the density functional theory (DFT) the adsorption behaviors of the three gases on intrinsic carbon nanotubes (CNTs) and Ni-CNTs were calculated. The adsorption energy, charge transfer, and molecular frontier orbital of the adsorption system were also analyzed. Results showed that the sensitivity of the CNT sensor to the three kinds of gases was in the following order: C_2_H_2_ > C_2_H_4_ > C_2_H_6_. Moreover, the doped Ni improved the sensor response, and the sensor response and gas concentration have a good linear relationship.

## 1. Introduction 

The special structures and properties of carbon nanotubes (CNTs) have attracted a great deal of attention of researchers at home and abroad since Iijima [1] introduced CNTs in 1991. CNTs have made a big difference in chemistry, physics, materials science, medicine, life sciences and other fields, which indicates that they have great potential application prospects [2,3].

CNTs are ideal materials for gas sensors because of their abundant pore structures, large surface-to-volume ratios, good conductivity and high surface activity. CNTs also exhibit strong adsorption and desorption capacity for gases [4,5,6,7,8,9,10]. Compared with traditional sensors, CNT gas sensors exhibit a fast response, high sensitivity, small size, and low working temperature [11,12].Transformers are expensive and significant electric components in power transmission and distribution systems. Their safety and stable operation is critical to the whole power system. In long-term operation, local overheating, discharges, and insulation paper aging in an oil-immersed transformer, may produce a certain amount of gases that dissolve in the transformer oil. Among them C_2_H_2_, C_2_H_4_, and C_2_H_6_ are important components of the oil-dissolved gases. In order to effectively predict, detect and recognize the internal faults and latent failures in transformers, it is necessary to detect the composition and content of oil-dissolved gases generated by these faults [13,14,15].

Gas chromatography (GC) is an effectively detection method which can accurately determine the concentrations of different gases [15,16]. However, during long time operation the performance of chromatograph columns also degrades, so that regular maintenance and recalibration are needed. GC monitoring systems are also costly and must usually be used in the laboratory, so online monitoring cannot be performed. In order to know the running status and insulation level of transformers at any time, an online monitoring device is needed. Gas sensing detection technology is the core of an online monitoring device, which directly affects the accuracy and stability of on-line monitoring systems together with its service life. Metal oxide sensors have high working temperatures, long response times and poor selectivity which make them unsuitable for detecting oil dissolved gases [17,18]. Thus, a study on a CNT gas sensor for the three gases of interest is of vital significance.

In this paper, the response of a CNT sensor was investigated by both experimental and theoretical calculations. Results show that the adsorptions between the three kinds of gases and the intrinsic CNTs are all weak physisorptions. The Ni-doped CNTs (Ni-CNTs) sensors strengthen the adsorptions, and show improved sensor responses to C_2_H_2_, C_2_H_4_, and C_2_H_6_.

## 2. Experimental Section

The CNTs used in this article were prepared using CVD, which is widely used in the synthesis of CNTs due to its many advantages such as low growth temperature, atmospheric pressure during the reaction process, low cost equipment and the production of products with high purity. The preparation method of the CNTs synthesis process and catalyst were similar to those described in [19]. Then a Ni-CNT gas sensor was developed. The sensing susceptibility of the sensor to C_2_H_2_, C_2_H_4_, and C_2_H_6_ was studied, and a plausible sensing mechanism was also analyzed.

### 2.1. Preparation of the Ni-CNT Sensor

The diameter of the CNTs used in this paper ranged from 20 nm to 30 nm, and their length ranged from 10 µm to 30 µm. The purity of the CNTs was more than 95%. Firstly, 0.1 g of CNTs was soaked in a 3:1 (v:v) solution of 98% sulfuric acid and 78% nitric acid, and then dispersed in an ultrasonic shaker for 60 min. Secondly, the solid phase was filtered and washed with deionized water several times until the solution became neutral, and then dried at 70 °C. The dark powder obtained is the mixed acid-modified CNTs.

**Figure 1:**
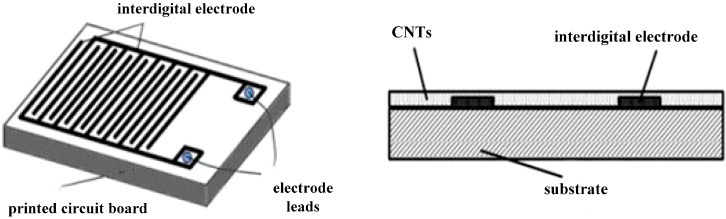
Sketch of the CNT sensor.

Taking appropriate amount of CNTs dissolved in anhydrous ethanol to prepare 1 mg/mL solutions. NiCl_2_·6H_2_O (20 mg) was dissolved in 50 mL of 1 mg/mL CNT suspension, and then the beaker containing the CNTs and NiCl_2_ was put in an ultrasonic bath for 90 min to obtain a uniform dispersed Ni-CNTs solution. Ni-CNTs thin films were prepared on the surface of interdigital electrodes (Figure 1) using coating drops and dried at 80 °C. This process was repeated to ensure a compact and smooth distribution of the sensing film, as shown in Figure 1.

### 2.2. Sensor Response Experiment

**Figure 2:**
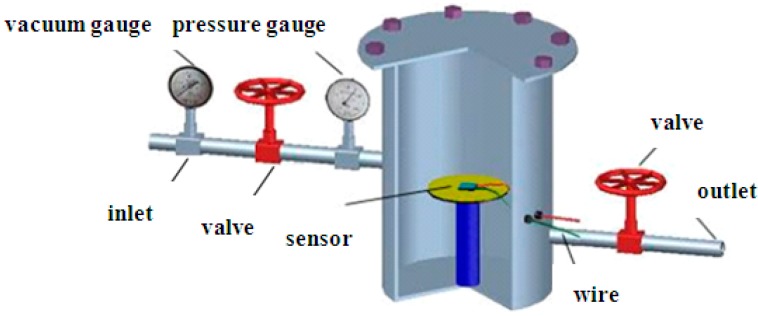
Schematic of the experimental setup.

The device for testing the gas-sensing properties is shown in Figure 2. The main part of the detection device is a steel chamber that is sealed by screws. Before the test, the pressure tightness of the device should be ensured.

First, the sensor was placed inside the chamber connected to an impedance analyzer through wires to record the changes in resistance, and then the cell was evacuated. Second, nitrogen gas was passed through the chamber until the resistance of the sensor stabilized. Then, different concentrations of the target gas species were passed into the sealed chamber through the inlet valve. The relative variation of the resistance was calculated as follows:

(1) R%=(R−R0)R0×100%

where *R* is the sensor resistance in relevant gas, and *R*_0_ is the sensor resistance in nitrogen gas. After each test the chamber of the device should be evacuated for the next test. All the experiments in this paper were performed at room temperature.

### 2.3. Experiment Result and Discussion

**Figure 3:**
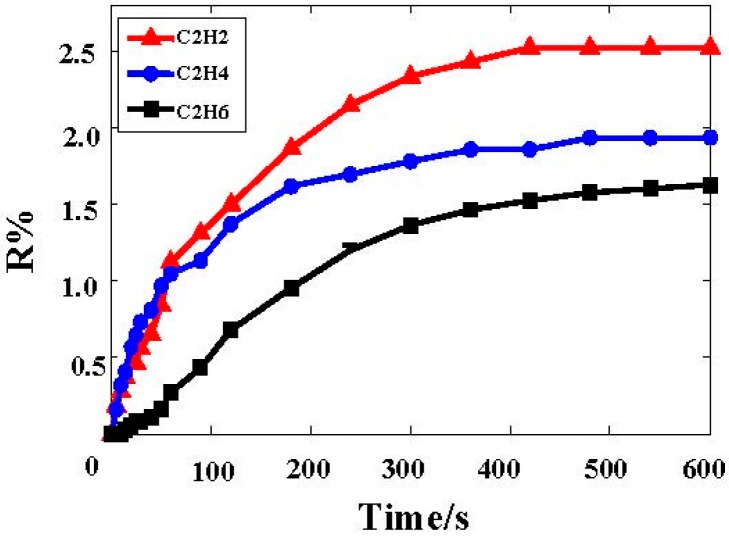
Ni-CNT sensor response to 10 µL/L C_2_H_2_, C_2_H_4_, and C_2_H_6_.

The responses of the Ni-CNTs gas sensor for the concentrations of 10 µL/L C_2_H_2_, C_2_H_4_, and C_2_H_6_ were tested using the method described above. The gas-sensitive response curves are shown in Figure 3, in which the horizontal axis represents time, while the vertical axis represents the change in resistance. In order to avoid accidental factors that affect the detection results, experimental results presented in this paper are the results of statistical analysis preformed on ten sensor samples instead of one set. The gas sensitivity in this paper is an average value. The calculated standard deviations of C_2_H_2_, C_2_H_4_, and C_2_H_6_ are 0.0374, 0.0288, 0.0275, respectively (data not shown).

Figure 3 shows that there is a sudden increase in the resistance of the Ni-CNT sensor when exposed to C_2_H_2_, C_2_H_4_, and C_2_H_6_ at first, and then the resistance becomes stable. It can be observed that the relative variations of the resistance for C_2_H_2_, C_2_H_4_, and C_2_H_6_ are nearly unchanged at 2.52%, 1.95%, and 1.61%, after 400 s. The results indicate that the Ni-CNTs sensor is most sensitive to C_2_H_2_ under the same concentration conditions compared with the other two kinds of gases. 

### 2.4. Sensor Response of Different C_2_H_2_ Concentrations

**Figure 4:**
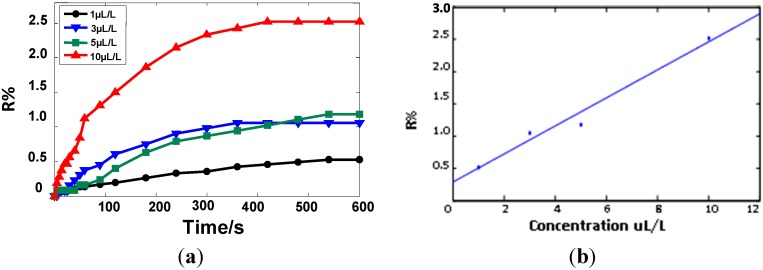
The gas-sensing properties of Ni-CNTs sensors to different concentrations of C_2_H_2_. (**a**) Gas-sensitivity to different concentrations of C_2_H_2_; (**b**) Liner fitting curve.

A standard critical value of the oil-dissolved gas in the transformer is 5 μL/L [20]. In order to meet the engineering requirements, the gas-sensitive response of C_2_H_2_ at concentrations of 1, 3, 5, and 10 µL/L were tested. The result is shown in Figure 4a. The relative change values of the resistance to 1, 3, 5, and 10 µL/L C_2_H_2_ are 0.52%, 1.05%, 1.18%, and 2.52%, respectively. With the increasing concentration, the relative change value of resistance increases, and the response time shortens. Figure 4b depicts the linear fit curve of the response and gas concentrations; the linear correlation coefficient *R*^2^ is 0.98. The result indicates that when the C_2_H_2_ gas concentration is between 1 and 10 µL/L, the relative change value of resistance satisfies a certain linear relationship with the gas concentration, which can be used to estimate the concentration of C_2_H_2_ gas. 

### 2.5. Reproducibility of Ni-Doped CNTs

**Figure 5:**
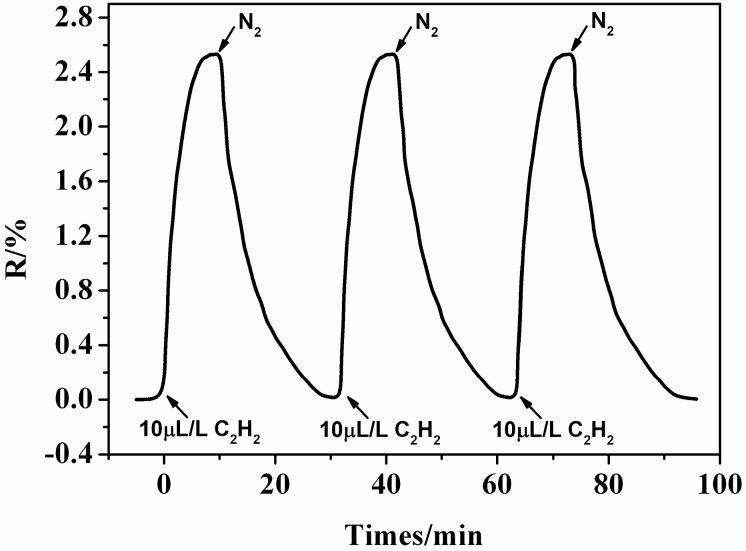
Reproducibility of Ni-doped CNTs sensor to 10 µL/L C_2_H_2_.

The sensor reliability is strongly dependent on the reproducibility exhibited by the sensor material. The reproducibility of the Ni-doped CNTs sensor was measured by repeating the response measurement three times. Tests were conducted according to the experimental steps described in Section 2.2. Pure N_2_ was used to accelerate the desorption of gas molecules. Figure 5 depicts the dynamic response transients for the Ni-doped CNTs sensors towards 10 µL/L C_2_H_2_ gas to illustrate the desorption and repeatability processes. Figure 5 shows that the response of the material is almost constant, confirming the reproducibility of sensor material. This suggests that the Ni-doped CNTs sensor can be used as a reusable sensing material for detecting oil-dissolved gases.

## 3. Theoretical Calculations 

The experimental results showed that the Ni-CNT sensor has different responses to oil-dissolved gases. To further understanding the sensing mechanism, the adsorption properties of the supports (CNTs and Ni-CNTs) to the gases were calculated and analyzed in a properly simplified model. Ni-substituted CNTs were constructed to simulate the sensor in our experiment.

### 3.1. Computational Details

**Figure 6:**
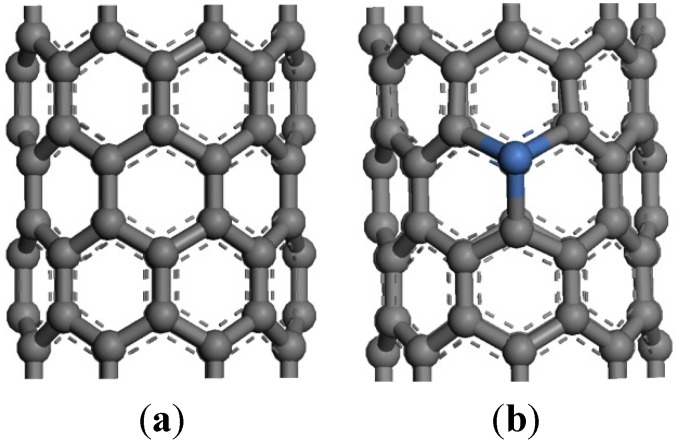
Structural model of CNTs and Ni-CNTs. (**a**) CNTs; (**b**) Ni-CNTs.

**Figure 7:**
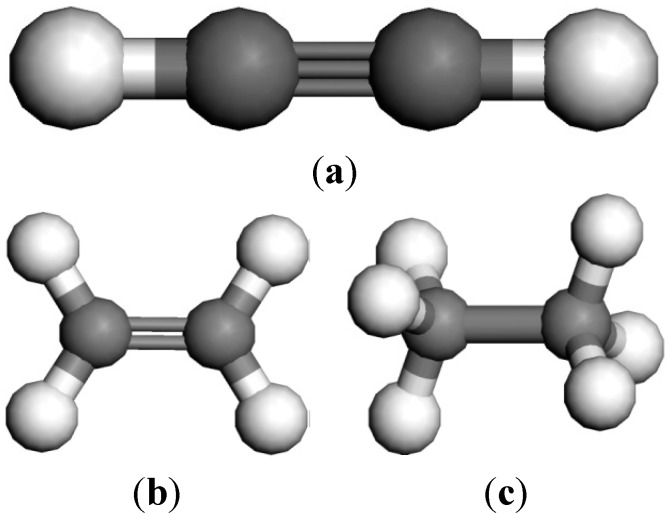
Structural model of oil-dissolved gases. (**a**) C_2_H_2_; (**b**) C_2_H_4_; (**c**) C_2_H_6_.

Fully optimized geometries and the properties of the systems were derived by DFT calculations in the generalized gradient approximation by using the DMol^3^ code with double-numerical polarized basis sets. The calculations were performed using the PBE [21] DFT. The structural models are shown in Figure 6 and Figure 7.

### 3.2. Results and Discussion

#### 3.2.1. Adsorption Energy and Charge Transfer

The interaction can be described by the adsorption energy *E_ads_*, which defined as follows:

(2) $$E_{ads}=E_{\left(gas/support\right)}-E_{\left(support\right)}-E_{\left(gas\right)}$$

where *E_(gas/support)_* is the total energy of a gas molecule adsorbed on the support surface, and *E_(support)_* and *E_(gas)_* are the total energies of the support and the gas molecule. If *E_ads_* < 0, the adsorption process is exothermic and spontaneous.

To obtain the charge distribution of the system, the charge transfer *Q_T_* was calculated by Mulliken population analysis and define d as the charge variation of the isolated gas molecule after the adsorption [22,23]. The adsorption energies are negative, which indicates that the adsorptions are exothermic. 

In the CNT system: The values of the energy released in adsorptions are as follows: C_2_H_2_ > C_2_H_4_ > C_2_H_6_, all of them are small (<0.6 eV). In addition, the charge transfers are very small and close to zero, which indicates that the gas molecules are physisorbed and not chemisorbed on the surface of CNTs, and the van der Waals interaction between the gas and the support is weak. The sensitivity of adsorption is as follows: C_2_H_2_ > C_2_H_4_ > C_2_H_6_.

In the Ni-CNT system: Table 1 shows that both the adsorption energy and charge transfer of the Ni-CNT system are significantly increased compared with the CNT system. The doped Ni effectively improved the electronic structure and sensitivities of CNTs. The sensitivity of the sensor to three gases is consistent with that of the CNTs. The adsorption of C_2_H_6_ is the weakest, and the adsorption energies of C_2_H_2_ and C_2_H_4_ are 8.7 and 4.6 times that of C_2_H_6_, respectively. The charge transfers of C_2_H_2_ and C_2_H_4_ are also higher than that of C_2_H_6_. The charge transfer of C_2_H_2_ is the highest, and the adsorption energy of C_2_H_2_ is nearly two times that of C_2_H_4_. Thus, Ni-CNTs have the highest sensitivity to C_2_H_2_, which is similar to that of the CNTs.

**Table 1:** Adsorption energy and charge transfer.

	*E_ads_* (eV)	*Q_T_* (e)
C_2_H_2_-CNTs	−0.3265	0.006
C_2_H_4_-CNTs	−0.2814	0.003
C_2_H_6_-CNTs	−0.0458	0.002
C_2_H_2_-Ni-CNTs	−1.7412	0.091
C_2_H_4_-Ni-CNTs	−0.9246	0.069
C_2_H_6_-Ni-CNTs	−0.1994	0.043

In summary, the sensitivity of the CNT sensor for the gases is as follows: C_2_H_2_ > C_2_H_4_ > C_2_H_6_, and the doped Ni can improve the sensor sensitivity.

#### 3.2.2. Frontier Molecular Orbital Analysis

The highest occupied molecular orbital (HOMO) energy and the lowest unoccupied molecular orbital (LUMO) energy of the three gas molecules and the supports were calculated according to the molecular orbit theory [24,25]. An analysis of HOMO and LUMO can determine whether charges can easily transform between gases and supports or not. *E_L-H_* is defined as follows:

(3) $$E_{L-H}=E_{LUMO}-E_{HOMO}$$

If *E_L-H_* is small, the energy charge needed to be transferred between the orbits is small, and the system with a good conductivity. The calculated *E_HOMO_*, *E_LUMO_*, and *E_L-H_* are listed in Table 2.

The *E_L-H_* of CNTs and Ni-CNTs are 0.6911 eV and 0.5470 eV, respectively. The doped Ni reduces the *E_L-H_* of 0.1441 eV that enhances the conductivity of the tube. After adsorption, the frontier orbital energies of the adsorption systems increased and *E_L-H_* also changed, there values are as followings: C_2_H_2_–Ni-CNTs < C_2_H_4_–Ni-CNTs < C_2_H_6_–Ni-CNTs. Thus, conductivities of the adsorption systems are as follows: C_2_H_2_–Ni-CNTs > C_2_H_4_–Ni-CNTs > C_2_H_6_–Ni-CNTs.

**Table 2:** Molecular frontier orbital energy and orbital energy differences.

	*E_HOMO_* (eV)	*E_LUMO_* (eV)	*E_L-H_* (eV)
CNTs	−4.5606	−3.8695	0.6911
Ni-CNTs	−4.9797	−4.4327	0.5470
C_2_H_2_–Ni-CNTs	−4.5906	−4.1606	0.4300
C_2_H_4_–Ni-CNTs	−4.6940	−4.2477	0.4463
C_2_H_6_–Ni-CNTs	−4.7593	−4.1933	0.5660

The conductivity change of the adsorption system in the process of Ni-CNTs absorbing C_2_H_2_ as well as the resistance change of the sensor sensing, C_2_H_2_ is the highest, while C_2_H_6_ is the lowest. This result indicates that the Ni-CNTs show the best sensitivity to C_2_H_2_ and the least sensitivity to C_2_H_6_. This finding is corresponding to gas sensing experiments.

## 4. Analysis of Experimental and Theoretical Results

In this paper, the research work includes theoretical and experimental studies on a Ni-CNT sensor for detecting oil-dissolved gases in a transformer. This research focuses on the gas-sensing response and mechanism. Gas sensing experimental results agreed with the simulation.

The gas molecules can be adsorbed on the surface of CNTs. Charge redistribution between the surfaces and the adsorbed molecules lead to changes in electronic structure and conductivity. A higher charge transfer results in a greater change in conductivity. The transfer charges calculated in this paper according to DFT are shown in Table 1. The transfer charges of C_2_H_2_ are the highest among these three gases, and the gas sensor showed the highest resistance variation to C_2_H_2_ (Figure 4). In addition, the orbital theory result coincides exactly with the charge transfer analysis and experimental results. Thus, in this paper, the theoretical analysis results are in agreement with the experiment results, and the sensitivities of Ni-CNTs to the three gases are as follows: C_2_H_2_ > C_2_H_4_ > C_2_H_6_. Besides, with increasing C_2_H_2_ concentration, the response time become shorter. The reason is that high gas concentrations can quickly adsorb on the surface of CNTs. In other words, adsorption rates improve in a shorter time.

Some studies have indicated that metal doping can effectively change the electronic structure and the conductivity of CNTs, therefore improving the gas sensing properties. Transition metals are d-electron rich and possess empty orbits, and the small gas molecules can bond strongly to the metal when adsorbed on the surface [26,27]. In this paper, nickel ions are the transition metal divalent cations used, which make nickel ions more accessible to the internal tubes in the capillary [28]. Besides, the surface active sites of the CNTs increase and the catalytic activity is greatly enhanced because of the coordination unsaturation of the nickel ion surface atoms. In general, the order of the chemical adsorption capacity of the transition metal to gas is as follows: O_2_ > C_2_H_2_ > C_2_H_4_ > CO > H_2_ > CO_2_ > N_2_. This order is consistent with the results of the article, namely, C_2_H_2_ > C_2_H_4_, which indicates that the doped Ni increases the chemical adsorption of the gas molecules. The adsorption of the gas molecules on the surface of the CNTs changed the surface barrier of the CNT gas-sensitive film, thereby significantly changing the electrical resistance of the sensor [5,29,30].

## 5. Conclusions

(1) The gases C_2_H_2_, C_2_H_4_, and C_2_H_6_ can be physically adsorbed on intrinsic carbon nanotubes, and the adsorption sensitivity is as follows: C_2_H_2_ > C_2_H_4_ > C_2_H_6_.
(2) Ni doped CNTs minimize the energy level difference and boost the conductivity of the CNTs. The adsorptions of the three gases became stronger, and the adsorption sensitivity of the Ni-CNTs was consistent with that of the CNTs.
(3) The surface active sites of the CNTs increased and catalytic activity was greatly enhanced because of the coordination unsaturation of the nickel ion surface atoms. The doped Ni improved the ability of the tube to adsorb gas molecules.
(4) When detecting low C_2_H_2_ concentrations (1 µL/L to 10 µL/L), the relative variation of the sensor resistance *R*% and the gas concentration meet a certain linear relationship, which indicates that the developed sensor can detect low gas concentrations.
